# Cognitive functioning in a cohort of high-grade glioma patients

**DOI:** 10.2478/raon-2023-0009

**Published:** 2023-06-21

**Authors:** Andreja Cirila Skufca Smrdel, Anja Podlesek, Marija Skoblar Vidmar, Jana Markovic, Jana Jereb, Manja Kuzma Okorn, Uros Smrdel

**Affiliations:** Department of Psycho-Oncology, Institute of Oncology Ljubljana, Ljubljana, Slovenia; Department of Psychology, Faculty of Arts, University of Ljubljana, Slovenia; Division of Radiotherapy, Institute of Oncology Ljubljana, Ljubljana, Slovenia; Faculty of Medicine, University of Ljubljana, Slovenia; University Medical Centre Ljubljana, Slovenia

**Keywords:** cognition, high grade glioma, IDH1 mutation, MGMT methylation

## Abstract

**Background:**

High grade gliomas are associated with cognitive problems. The aim of the study was to investigate cognitive functioning in a cohort of patients with high grade glioma, according to isocitrate dehydrogenase (IDH) and methyl guanine methyl transferase (MGMT) status and other clinical characteristics.

**Patients and methods:**

The patients with the high-grade glioma treated in Slovenia in given period of time were included in study. Postoperatively they completed neuropsychological assessment consisting of Slovenian Verbal Learning Test, Slovenian Controlled Oral Word Association Test, Trail Making Test Part A and B and self-evaluation questionnaire. We analysed results (z-scores and dichotomized results) also according to IDH mutation and MGMT methylation. We examined differences between groups using T-test, Mann-Whitney U, χ^2^ and Kendall's Tau tests.

**Results:**

Out of 275 patients in the cohort, we included 90. Forty-six percent of patients were unable to participate due to poor performance status and other conditions related to tumour. Patients with the IDH mutation were younger, with better performance status, larger proportions of grade III tumours and MGMT methylation. In this group cognitive functioning is significantly better in the domains of immediate recall, short delayed recall and delayed recall, and in the fields of executive functioning and recognition. There were no differences in cognitive functioning in regard to MGMT status. Grade III tumours were associated with more frequent MGMT methylation. Self-assessment proved week tool, associated only with immediate recall.

**Conclusions:**

We found no differences in cognitive functioning according to MGMT status, but cognition was better when IDH mutation was present. In a cohort study of patients with high-grade glioma, almost half were unable to participate in a study, which points to an overrepresentation of patients with better cognitive functioning in the research.

## Introduction

Malignant gliomas are group of aggressive brain tumours, comprising anaplastic astrocytoma (Grade III), anaplastic oligodendrogliomas (Grade III), anaplastic astrocytoma (Grade IV) and glioblastomas (Grade IV).^1^ Anaplastic gliomas are still some of the most challenging tumours for patient and caregivers, but also for the therapist. Although there are some cautious advances in this field, there is still a grim outlook for the patients. As a number of patients is ill responding to treatment there are efforts for identifying those who respond well and those who would benefit from a change in treatment strategy.

Genetic and epigenetic markers like isocitrate dehydrogenase (IDH) mutations, loss of heterozygosity of 1p/19q(LoH 1p/19q), and methyl guanine methyl transferase (MGMT) promoter methylation have recently helped to stratify patients, removing the mixed histology like anaplastic oligoastrocytoma and introducing Grade IV astrocytoma. In IDH1 mutated patients’ survival was markedly longer, as is in anaplastic oligodendrogliomas (with LoH 1p/19q). Prior to widespread genetic testing, it was already clear that patients harbouring methylation of MGMT gene promoter fare better comparing to those without.^2,3,4^

Brain tumours are also associated with impaired cognitive functioning, due to tumour alone but also due to treatment. Cognitive impairment can manifest already at the time of diagnosis; the prevalence of cognitive deficits varies from 60 to 85% in different studies.^5^ The most common are in the fields of verbal memory, executive functioning, psycho-motor speed, but also attention and language.^6,7,8,9^

Cognitive functioning is also one of the prognostic factors for survival. Early findings suggested that cognitive decline is preceding radiological progression, which was not confirmed by all studies.^10,11,12^ Further studies confirmed cognitive impairment as independent prognostic factor in newly diagnosed patients, both at baseline and in the period after surgery.^13,14,15,16^

It was shown that IDH1 mutation (IDH1-mut) is not only an important prognostic factor, but it is also associated with better cognitive functioning. Many studies have shown that cognitive functioning is better in IDH1-mut patients when compared with IDH1-wildtype (IDH1-wt) patients.^17,18,19^

Among possible causes of the better cognitive functioning of patients with IDH1-mut is brain plasticity which could be affected negatively by the greater tumour growth rate in IDH1-wt tumours, while remaining intact in less invasive IDH1-mut tumours. Preserved cognitive functioning might also be related to the tumour microenvironment, with more pronounced lymphocyte infiltration and programmed death-ligand 1 (PD-L1) expression in IDH1-wt tumours, or even to differences at the synaptic level.^20^

The effect and possible role of the MGMT methylation status in patients’ cognitive functioning is even less clear. Most clinical studies focus on investigating patients with MGMT promoter methylation (MGMT-met), with cognitive function as a secondary outcome.^21^ According to one study, the absence of MGMT promoter methylation (MGMT-unmet) predicts greater cognitive deficit when patients are treated with radiochemotherapy.^22^ The MGMT-met therefore can be considered a predictive marker for development of cognitive impairment, but further research about its role in cognitive functioning as well as the prognosis needed. Here the challenge is the frequent overlap of MGMT promoter methylation with the IDH1 mutation, which, in conjunction with relatively small number of patients in the high-grade glioma studies, presents difficulties in statistical analysis.

In our study, we examined how the expression of IDH1 mutation and MGMT promoter methylation are linked to the cognitive functioning following the operative treatment in the cohort of all Slovene Grade III and Grade IV glioma patients.

## Patients and methods

### Patients

We analysed the cohort of patients with high grade glioma, treated between March 2019 and December 2021. Their diagnoses (anaplastic astrocytoma, anaplastic oligodendroglioma or glioblastoma) were histological confirmed. Patients were operated in either of the two neurosurgical departments in Slovenia, then they were referred to Institute of Oncology Ljubljana for evaluation regarding the initiation of radiochemotherapy.

At the referral, they consented to be enrolled in the study. Exclusion criteria were histology other than gliomas WHO III/IV, Karnoffsky performance status less than 70% and inability to undergo evaluation (e.g., marked dysphasia). To be included in the study, they had to be 18 years old or older.

The following data were obtained from the medical documentation: age, sex, date of diagnosis, localization of the tumour, type of surgery, extent of surgery, radiotherapy parameters, systemic therapy, use of corticosteroids, histological, genetic and epigenetic characteristics of tumours. All patients also had a molecular and genetic analysis of the tumour tissue performed, so the IDH1 mutations and MGMT promoter methylation status were determined.

### Cognitive functioning

To asses cognitive functioning, we used psychometric tests in the domains of verbal memory (Slovenian Verbal Learning Test – TBU, measuring immediate recall, short recall, delayed recall and recognition of distracters)^23^, verbal fluency (Slovenian Controlled Oral Word Association Test –SCOWA)^24^, psycho-motor speed (Trail Making Test, Part A – TMT A), executive functions (Trail Making Test, Part B – TMT B)^25^, in accordance with the recommendations for use in the studies concerning cognitive functioning of cancer patients.^26^ The patients also self-evaluated their cognitive functioning on a 0–10 scale (0 = without problems, 10 = extremely intensive problems present).

### Statistical analysis and ethical consideration

We used descriptive statistics with the means values and standard deviation for the demographic data. The correlation between variables was tested with Pearson's t test or Spearman's rho test.

For each cognitive test, the test scores were analysed either as standardized (z-scores) or as a dichotomized variable: no impairment present (z > −1.5 below the mean of the control group) *vs*. impairment observed (the patient had a z-score lower than −1.5 or was unable to perform the test at all). At the individual level, we analysed the percentage of impaired patient's results.

We next compared groups of patients with different IDH1 statuses and MGMT methylation, using either a t-test or Mann-Whitney U-test (in case that Kolmogorov-Smirnov test of normality showed statistically significant departure from normality) for interval variables, a χ^2^ test for categorical variables and ordinal variables with Kendall's Tau test. All hypotheses were tested at a 5-percent alpha error rate.

We used the statistical program SPSS, to calculate the power of the test we used G*Power 3.1.9.7.

Written informed consent was obtained from all the patients before the inclusion in the clinical trial. The study was approved by the Institutional Review Board of the Institute of Oncology Ljubljana and by The National Medical Ethics Committee of the Republic of Slovenia (Approval number 0120-393/2018/10, date 12/12/2018) and was carried out according to the Declaration of Helsinki.

## Results

### Demographics and tumour characteristics

**FIGURE 1.**
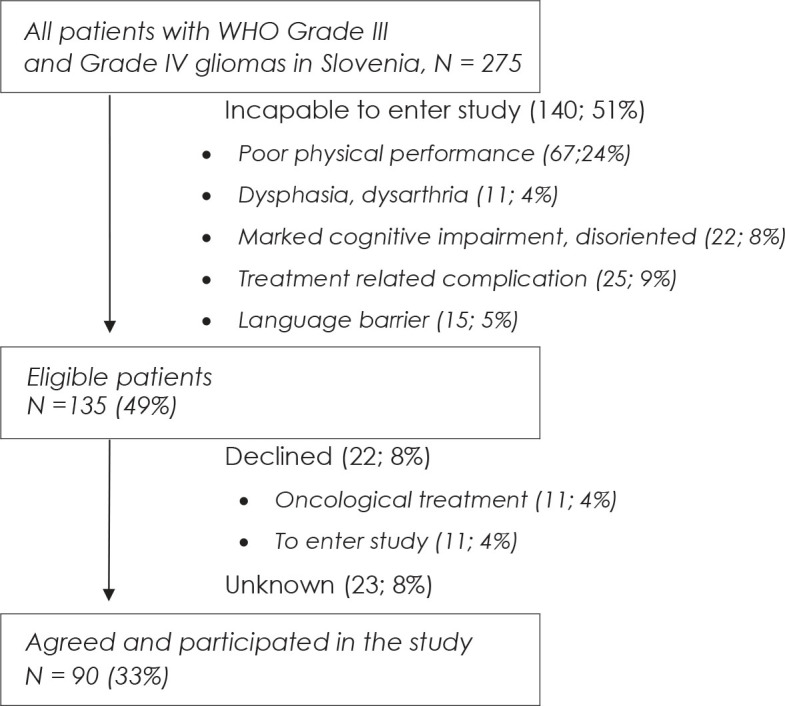
Recruitment protocol, N = 275

At the time the research was performed, 275 patients were diagnosed with glial tumour. Of those, 90 patients were recruited into the study, representing 33% of all patients. Figure 1 shows reasons for patients entering and not entering the study as recorded by oncologists at the time of the first consultation. Of the 51% of patients incapable of participating, in the study the major reason was poor performance status, followed by other tumour and treatment related impairments, representing 46% of ineligible patients, the other common issue was language barrier.

Participating patients were 30 to 84-year-old (median [M] = 58.78 years, standard deviation [SD] = 11.31 years). There were more males than females (57 *vs*. 33). On average, female patients were older (M = 57.67 years, SD = 9.35 years, 39–74 years) than male patients (M = 59.42 years, SD = 12.33 years, 30–84 years), but the age difference was not statistically significant (p = 0.481).

Of all 90 patients, 78 had Grade IV tumour (all classified as glioblastoma), 8 had anaplastic astrocytoma, and 4 had anaplastic oligodendroglioma. Fifteen patients had IDH1 mutation (7 anaplastic oligodendroglioma, 4 anaplastic astrocytoma, and 4 glioblastoma). MGMT promoter was methylated in 36 patients (4 anaplastic oligodendroglioma, 5 anaplastic astrocytoma, and 27 glioblastoma), whereas in 1 glioblastoma, 2 anaplastic astrocytomas and 1 anaplastic oligodendroglioma we couldn’t determine the methylation status.

Patients with Grade III tumours had a statistically better performance status – the Karnoffsky performance status was 70 in 2 (17%) patients, 80 in 2 (17%) patients, 90 in 8 (67%) patients with Grade III tumours – when compared with patients with Grade IV tumours (in these group, the Karnoffsky performance status was 70 in 30 patients, 80 in 35 patients, 90 in 10 patients, and 100 in 3 patients; *U* = 251.000, *z* = −2.75, *p* = 0.006). Compared to the group of patients with Grade III tumours, the group with Grade IV tumours had a statistically significantly higher percentage of IDH1-mut tumours (73% *vs*. 5%, *χ^2^*(1) = 56.077, *p* < 0.001, *V* = 0.789, *1–b* = 0.987) and MGMT promoter methylations (60% *vs*. 36%, *χ^2^*(1) = 7.067, *p* = 0.008, *V* = 0.280, *1–b* = 0.756).

Table 2 shows the structure of the sample according to the expression of IDH1 mutation and MGMT methylation. Five patients had both genetic markers expressed, two thirds of patients with IDH1-mut also had MGMT-met expressed, and among patients with expressed methylation, IDH1-mut tumours were present in 27% of patients. The difference in the proportion of MGMT methylation in the IDH1-mut and IDH1-wt groups was statistically significant, *χ^2^*(1) = 5.333, *p* = 0.021, *V* = 0.243, *1–b* = 0,82).

**TABLE 1.** Demographic and medical data

**Variable**	**Levels**	**f (%)**	**Descriptive statistics**
Gender	Male	57 (63%)	
	Female	33 (37%)	
Age	< 50 years	17 (19%)	M = 58.78, SD = 11.31 min = 30, max = 84
	50–70years	60 (67%)	
	> 70 years	13 (14%)	
Education	≤9 years	14 (16%)	
	10–13 years	48 (53%)	
	14–19 years	26 (29%)	
	≥20 years	2 (2%)	
Tumour grade	Grade III	12 (13%)	
	Grade IV	78 (87%)	
Tumour location	Frontal	34 (38%)	
	Parietal	21 (23%)	
	Temporal	26 (29%)	
	Occipital	4 (4%)	
	central	3 (3%)	
	Diffuse	2 (2%)	
Hemisphere	Right	38 (42%)	
	Left	44 (49%)	
	Both	8 (9%)	
Surgery type	Biopsy	11 (12%)	
	Reduction	49 (54%)	
	Gross tumour resection	30 (33%)	
Karnoffsky performance status	70	32 (36%)	
	80	37 (41%)	
	90	18 (20%)	
	100	3 (3%)	
Corticosteroids (Yes/no, mg)	Yes	66 (73%)	M = 5.55, SD = 4.73 min 0, max 24
	No	24 (27%)	
Radio-chemotherapy (intention to treat)	Yes	90 (100%)	
	No	0 (0%)	
Time to beginning of adjuvant treatment (in weeks)	≤ 6 weeks	60 (67%)	M = 5.98, SD = 2.25 min 3, max 15
	≥ 7 weeks	30 (33%)	
Epilepsy	Yes	27 (30%)	
	No	63 (70%)	
IDH1 mutation	IDH1 mutation	15 (17%)	
	IDH1 wild type	75 (83%)	
MGMT methylation*	Yes	36 (42%)	
	No	50 (58%)	

IDH1 = isocitrate dehydrogenase 1; M = median; MGMT = methyl guanine methyl transferase; SD = standard deviation

**TABLE 2.** Number of patients with different combinations of IDH1 mutation expression and methyl guanine methyl transferase (MGMT) methylation

	**MGMT-met**	**MGMT-unmet**	**Total**
**IDH1-mut**	5	10	15
**IDH1-wt**	49	26	75
**Total**	54	36	90

IDH1-mut = isocitrate dehydrogenase 1 mutation; IDH1-wt = isocitrate dehydrogenase 1 wildtype; MGMT-met = methyl guanine methyl transferase promoter methylation; MGMT-unmet = methyl guanine methyl transferase absence of promoter methylation

### Cognitive functioning

The overview of cognitive evaluation is presented in Table 3. The achievements were impaired (z <−1.5) in a large proportion of the patients, especially in the field of short recall, executive functions and psycho-motor speed. The impairment was least frequent in the field of recognition and verbal fluency. The results remained similar, regardless of accounting only those who were capable of completing a specific test or the sample as whole – the biggest difference between these two analysis methods is on the TMT A and TMT B tests.

**TABLE 3.** Descriptive statistics for standardized test scores (z–values) and proportion of impaired patients in psychological cognitive functioning tests

**Domain**	**Test**	**% impaired / all**	**N**	**% impaired / capable**	**Mean z score**	**SD of z scores**
Visual – motor speed	TMT A	68	68	57	2.89	3.57
Executive function	TMT B	78	46	59	2.80	2.94
Verbal fluency	SCOWA	47	81	41	−1.21	0.88
Memory						
immediate recall	SVLT-ir	64	80	60	−1.83	1.11
short delayed recall	SVLT-sr	79		76	−2.05	1.31
delayed recall	SVLT-dr	63		58	−1.92	1.35
recognition	SVLT-recog	60		52	−2.73	3.36

SCOWA = Slovenian Controlled Oral Word Association Test; SVLT = Shiraz Verbal LearningTest; TMT A = Trail Making Test, Part A; TMT B = Trail Making Test, Part B

We examined on how many out of 7 tests the patients had an impaired test score (*z* < −1.5 or unable to finish the test) and found that 11 patients (12%) had 0–1 impaired test score, 26 (29%) patients had 2–4 impaired test scores, and 53 (60%) patients had more than 5 impaired test scores.

On the 10-point self-evaluation scale of cognitive functioning the mean rating was 3.66, SD = 2.81, min = 0, max = 10; 48 (53%) patients selected rating 0–3, 23 (26%) selected rating 4–6, and 19 (21%) selected a rating higher than 7. Correlations between self-assessment and individual tests of cognitive functioning show that self-assessment is weakly but statistically significantly related only to immediate recall (*r* = −0.280, *df* = 79, *t* = 2,57, *p* = 0.012).

The cognitive functioning in any of the measured fields was not statistically significantly affected by sex, surgery type and the presence or absence of seizures. The test scores did, however, differ with regard to age, education, and performance status. Age was statistically significantly related to participant's results in the field of verbal fluency (*r* = −0.278, *t* = −2.55, *df* = 79, *p* = 0.012), immediate recall (*r* = −0.409, *t* = −3.96, *df* = 79, *p* < 0.001), short delayed recall (*r* = −0.388, *t* = 3.72, *df* = 79, *p* < 0.001) and delayed recall (*r* = −0.333, *t* = 3.12, *df* = 79, *p* = 0.003).

Education was significantly related to results in the fields of verbal fluency (*r*_s_ = 0.381, *t* = 3.66, *df* = 79, *p* < 0.001), immediate recall (*r*_s_= 0.334, *t* = 3.13, *df* = 79, *p* = 0.002), short delayed recall (*r*_s_ = 0.285, *t* = 2.63, *df* = 79, *p* = 0.010), delayed recall (*r*_s_ = 0.265, *t* = 2.42, *df* = 80, *p* = 0.017) and recognition (*r*_s_ = 0.264, *t* = 2.42, *df* = 79, *p* = 0.018).

Performance status was significantly related to immediate recall (*r*_s_ = 0.280, *t* = 2.57, *df* = 79, *p* = 0.012) and delayed recall (*r*_s_ = 0.296, *t* = 2.74, *df* = 79, *p* = 0.008).

We analysed the disease and demographic data and the results of psychological tests with regard to IDH1 mutation. Compared to IDH1-wt patients, patients with tumours harbouring IDH1-mut were statistically significantly younger, had better performance status and were more likely to have Grade III tumour and MGMT promoter methylation (Table 4). They functioned better in the field of verbal memory (had a better performance in immediate recall, short delayed recall and delayed recall, measured either with z-scores or as dichotomised test scores) and in the field of executive functioning (measured with dichotomised test scores) (Table 5).

**TABLE 4.** Patient characteristics, regarding isocitrate dehydrogenase 1 (IDH1) mutation

	**IDH1-wt (N = 75)**	**IDH1-mut (N=15)**	**Result of the statistical test and effect size**
Age mean (min/max/SD)	61.50 (31 / 84 / 9.21)	38.75 (30 / 67 / 3.86)	*t* = −5.97, *df* = 88, *p*< 0.001
Sex (female / male)	29 / 46	4 / 11	*χ^2^*(1) = 0.775, *f* = −0.93, *p* = 0.379
Education level (≤ 9 years /10–13 years / 14–19 years / ≥ 20 years)	11 / 40 / 22 / 2	3 / 8 / 4 / 0	*τb*(3) = −0.06, *z* = −0.83, *p* = 0.547
KPS (70/80/90/100)	31 / 34 / 7 / 3	1 / 3 / 11 / 0	*τb*(3) = 0.403, *z* = 18.95, *p* < 0.001
WHO grade (III / IV)	1 /74	11 /4	*χ^2^*(1) = 56.08, *f* = −0.789, *p* < 0.001
Corticosteroids mg (min/max/SD)	2 (0 / 16 / 6)	5 (0 / 24 / 4.5)	*t* = −1.16, *df* = 88, *p* = 0.251
biopsy/reduction/gross tumour resection	9 / 44 / 22	2 / 5 / 8	*χ^2^*(2) = 3.65, *V* = 0.210, *p* = 0.161
Tumour location (frontal / temporal / parietal / occipital / diffuse / central)	26 / 20 / 21 / 4 / 2 / 2	8 / 1 / 5 / 0 / 1 / 0	NA
Hemisphere (right / left / both)	21 / 27 / 6	17 / 17 / 2	*χ^2^*(2) = 1.14, *V* = 0.113, *p* = 0.566
MGMT (yes / no)	10 / 5	26 /49	*χ^2^*(1) = 5.33, *f* = 0.24, *p* = 0.021

IDH1-mut = isocitrate dehydrogenase 1 mutation; IDH1-wt = isocitrate dehydrogenase 1 wild type; KPS = Karnoffsky performance status; MGMT = methyl guanine methyl transferase; NA = not available; SD = standard deviation

**TABLE 5.** Cognitive functioning regarding to isocitrate dehydrogenase 1 (IDH1) mutation

	**IDH1–wt**		**IDH-mut**		**Result of the statistical test**	**IDH1–wt**	**IDH1-mut**	**Result of the statistical test**
	
	**N**	**Mean Z score (SD)**	**N**	**Mean Z score (SD)**		**% of impaired**	**% of impaired**	
TMT A	53	3.12^1^ (3.84)	15	2.06 (2.23)	*U* = 345.000, *z* = −0.77, *df* = 66, *p*= 0.437	72	46	*χ^2^*(1) = 3.67, *V* = 0.20, *p* = 0.055
TMT B	33	2.82^1^ (3.11)	13	2.76 (2.57)	*U* = 221.500, *z* = −0.17, *df* = 66, *p* = 0.864	82	60	*χ^2^*(1) = 3.87, *V* = 0.21, *p* = 0.050
SCOWA	66	−1.28 (0.87)	15	−0.86 (0.84)	*t* = −1.69, *df* = 79, *p* = 0.095	51	27	*χ^2^*(1) = 2.89, *V* = 0.18, *p* = 0.089
SVLT-ir	65	−1.98 (1.06)	15	−1.15 (1.12)	*t* = −2.729, *df* = 78, *p* = 0.008	71	33	*χ^2^*(1) = 7.60, *V* = 0.29, *p* = 0.006
SVLT-sr	65	−2.20 (1.29)	15	−1.37 (1.22)	*t* = −2.25, *df* = 78, *p* = 0.027	84	53	*χ^2^*(1) = 7.06, *V* = 0.28, *p* = 0.008
SVLT-dr	65	−2.14 (1.27)	15	−0.98 (1.34)	*t* = −3.13, *df* = 78, *p* =0.002	69	33	*χ^2^*(1) = 6.98, *V* = 0.28, *p* = 0.008
SVLT-recog	65	−3.06^1^ (3.54)	15	−1.24 (1.91)	*U* = 611.000, *z* = −2.25, *df* = 78, *p* = 0.023	29	26	*χ^2^*(1) = 3.00, *V* = 0.18, *p* = 0.083

1 the distribution is significantly non-normal

IDH1-mut = isocitrate dehydrogenase 1 mutation; IDH1-wt = isocitrate dehydrogenase 1 wildtype; SCOWA = Slovenian Controlled Oral Word Association Test; SD = standard deviation; SVLT = Shiraz Verbal Learning Test; SVLT-dr = SVLT delayed recall; SVLT-ir = SVLT immediate recall; SVLT-recog = SVLT recognition; SVLT-sr = SVLT short delayed recall; TMT A = Trail Making Test, Part A; TMT B = Trail Making Test, Part B

Patients with IDH1-mut had on average a statistically significantly lower number of impaired tests results than patients with IDH1-wt (M = 2.93, SD = 2.25*vs*. M = 4.93, SD = 1.99; *U* = 286.000, *z* = −3.04, *p* = 0.002). 5 (33%) patients with IDH1-mut tumours and 6 (8%) patients without IDH1-mut had at most one impaired result on cognitive tests; impaired results on 2–4 tests had 5 (33%) *vs*. 21 (28%). Impaired scores on more than 5 tests had 5 (33%) *vs*. 48 (64%).

We found no differences between groups with regard to self-evaluation of cognitive functioning problems; the mean rating was 3.60, SD = 2.81, in patients with IDH1-mut tumours *vs*. 3.67, *SD* = 2.95 in patients with IDH1-wt (*U* = 579.000, z = 0.18, *p* = 0.857); 40 (53%) patients with IDH1-wt tumour gave a self-assessment of 0–3 *vs*. 8 (53%) patients with IDH1-mut, score 4–6 was given by 18 (24%) *vs*. 5 (33%) patients and a score above 7 17 (23%) *vs*. 2 patients (13%).

We also compared demographic characteristics in patients with and without MGMT promoter methylation. The patients differed in the tumour grade. Despite the predominance of Grade IV tumours in our sample, the methylated phenotype was more prevalent in Grade III patients (25% *vs*. 5%, *χ*^2^(1) = 7.067, *V* = 0.28, *1 − β* = 0.757, *p* = 0.008). In all other demographic characteristics, the groups were comparable.

In the cognitive functioning, there were no differences in mean z-scores or dichotomized test scores between patients with methylated and unmethylated promoter MGMT (Table 7).

**TABLE 6.** Patient characteristics regarding methyl guanine methyl transferase (MGMT) methylation

	**MGMT-unmet (N=54)**	**MGMT-met (N = 36)**	**Result of the statistical test and effect size**
Age mean (min/max/SD)	58.94 (31 / 84 / 10.42) 1	58,53 (30 / 78 / 12.67)	*t* = −0.17, *df* = 88, *p* = 0.86
Sex (female / male)	19 / 35	14 / 22	*χ^2^*(1) = 0.13, *f* = 0.04, *p* = 0.721
Education level (≤9 years /10–13 years / 14–19 years / ≥ 20 years)	8 / 19 / 11 / 0	9 / 29 / 15 / 2	*τb* (3) = −0.03, *z* = −1.41, *p* = 0.776
KPS (70/80/90/100)	20 / 23 / 9 / 2	12 / 14 / 9 /1	*τb* (3) =0.06, *z* = 2.82, *p* = 0.541
WHO grade (III / IV)	3 / 51	9 / 27	*χ^2^*(1) = 7.07, *f=* −0.28, *p* = 0.008
Corticosteroids mg (min/max/SD)	5.67 (0 /24 / 5,04)	5.48 (0 / 16 / 4.22)	*t* = 0.18, *df* = 88, *p* = 0.857
Biopsy/reduction/gross tumour resection	9 / 31 / 14	2 / 18 / 16	*χ^2^*(2) = 4.62, *V* = 0.23, *p* = 0.099
Tumour location (frontal / temporal / parietal / occipital / diffuse / central)	26 / 20 / 21 / 4 / 2 / 2	8 / 1 / 5 / 0 / 1 / 0	NA
Hemisphere (right / left / both)	28 / 40 / 7	10 / 4 / 1	*χ^2^*(2) = 4.46, *V* = 0.223, *p* = 0.107
IDH1 (yes / no)	5 / 49	10 / 26	*χ^2^*(1) = 5.33, *f* = 0.24, *p* = 0.021

IDH1 = isocitrate dehydrogenase 1; MGMT-met = methyl guanine methyl transferase promoter methylation; MGMT-unmet = methyl guanine methyl transferase absence of promoter methylation; KPS = Karnoffsky performance status; SD = standard deviation

**TABLE 7.** Cognitive functioning regarding methyl guanine methyl transferase (MGMT) methylation

	**MGMT-met**		**MGMT-unmet**		**Result of the statistical test**	**MGMT-met**	**MGMT –unmet**	**Result of the statistical test**
	
	**N**	**Mean Z score (SD)**	**N**	**Mean Z score (SD)**		**% of impaired**	**% of impaired**	
TMT A	26	2.67 (3.01)	42	3.03^1^ (3.90)	*U* = 530.000, *z* = −0.20, *df* = 67, *p* = 0.840	69	66	*χ^2^*(1) = 0.08, *V* = 0.03, *p* = 0.782
TMT B	20	3.22 (2.30)	26	2.48^1^ (3.37)	*U* = 334.000, *z* = 1.64, *df* = 45, *p* = 0.101	86	74	*χ^2^*(1) = 1.88, *V* = 0.14, *p* = 0.170
SCOWA	33	−1.25 (0.87)	48	−1.18^1^ (0.89)	*U* = 756.000, *z* = −0.35, *df* = 70, *p* = 0.729	50	44	*χ^2^*(1) = 0.27, *V* = 0.05, *p* = .605
SVLT-ir	32	−1.85 (1.27)	48	−1.82^1^ (1.01)	*U* = 771.000, *z* = 0.03, *df* = 78, *p* = 0.975	64	67	*χ^2^*(1) = 0.01, *V* = 0.01, *p* = 0.928
SVLT-sr	32	−2.03 (1.32)	48	−2.06 (1.32)	*t* = 0.09, *df* = 78, *p* = 0.926	75	81	*χ^2^*(1) = 0.54, *V* = 0.08, *p* = 0.460
SVLT-dr	32	−1.98 (1.52)	48	−1.89 (1.25)	*t* = −0.30, *df* = 78, *p* = 0.763	66	61	*χ^2^*(1) = 0.29, *V* = 0.06, *p* = 0.592
SVLT-recog	32	−2.731 (4.24)	48	−2.72^1^ (2.71)	*U* = 784.000, *z* = 0.828, *df* = 78, *p* = 0.407	58	61	*χ^2^*(1) = 0.07, *V* = 0.03, *p* = 0.792

1 the distribution is significantly non-normal

MGMT-met = methyl guanine methyl transferase promoter methylation; MGMT-unmet = methyl guanine methyl transferase absence of promoter methylation; SD = standard deviation; SVLT = Shiraz Verbal Learning Test; SVLT-dr = SVLT delayed recall; SVLT-ir = SVLT immediate recall; SVLT-recog = SVLT recognition; SVLT-sr = SVLT short delayed recall; TMT A = Trail Making Test, Part A; TMT B = Trail Making Test, Part B

There were no statistically significant differences in self-evaluation of cognitive functioning problems; the mean number of impaired results were 4.50 (*SD* = 1.90) in patients with MGMT un-methylated tumours *vs*. 4.29 (*SD* = 1.75), *U* = 1100.000, *z* = 1.51, *p* = 0.251.

There were also no differences in the number of tests in which patients achieved an impaired result (*U* = 1052.500, *z* = 0.67, *p* = 0.501). With the mean 4.69 (*SD* = 2.31) and 4.54 (*SD* = 2.07) had 5 (14%) patients with MGMT methylated tumours and 6 (11%) patients MGMT unmethylated tumours at most one impaired result, 2–4 impaired results had 9 (25%) *vs*. 17 (31%) patients, and on more than 5 tests the results were impaired in 22 (61%) *vs*. 31 (57%) patients.

## Discussion

High-grade glioma patients are experiencing a number of cognitive functioning problems. In our study we focused on the period following the surgical treatment and before commencement of systemic treatment.

The majority of cognitive problems we found were in the fields of executive functions, visual-motor speed and verbal memory, especially immediate and short delayed recall, as well delayed recall. There were the least problems in the field of verbal fluency, but even here more than 40% of patients had an impaired result. Among participating patients, only 12% had an impaired result in up to one measured field, while 60% had impaired results in the majority of the measured domains.

The analysis of cognitive test scores expressed as z-values gave conclusions comparable to the ones obtained with the analysis of dichotomized scores. The use of dichotomized scores enabled us to also include in the analyses the results of patients who were unable to complete some tests and so avoiding the overrepresentation of patients with better cognitive functioning in the analyses.

These results are in accordance with other studies examining cognitive functions in high-grade glioma patients, but it is noticeable that in our study the proportion of patients presenting with the “impaired” result is higher, possibly due to the fact that our study included the entire cohort of high-grade glioma patients. When comparing the results of different studies, it is necessary to consider the use of different criteria for impairment, with the otherwise dominant criterion *z*< −1.5.^5^

In comparison with IDH1-wt patients, patients with IDH1-mut (17%), were significantly younger, had better performance status and more often they had Grade III tumour. This is in line with previous studies.^27^

Additionally, the cognitive functioning of patients with IDH1-mut was statistical significantly better in verbal memory and executive functions. Immediate recall, short-delayed and long-delayed recall differ statistically significantly in the analysis of interval variables as well as in the analysis of dichotomized variables. Executive functions measured with the TMT B test only in the analysis of dichotomized variables, which may be the result of the fact that a larger proportion of patients were unable to complete this test, therefore, they are not included in the analysis of interval variables. Patients with IDH1-mut tumours achieved impaired results on significantly lower number of tests. These findings are in line with findings of the previous studies.^8,19^

According to the MGMT promoter methylation status, the groups did not differ statistically significantly in demographic data. A statistically significant difference was found in the expression of MGMT methylation according to the grade of the tumour (75% patients with grade III *vs*. 34% with grade IV).^28^ We did not find differences in any of the analysed fields of cognitive functioning and also not in the number of tests in which patients achieved an impaired result.

We intended to include all patients with the diagnosis of high-grade glioma in the observed period in Slovenia. Given that, after surgery in one of the two centres in Slovenia, all newly diagnosed patients with gliomas are referred to our institution for evaluation regarding further treatment; it gives us an insight into the entire population of patients with glioma. Data collected on the entire cohort of patients revealed that a large proportion of high-grade glioma patients is unable to participate in the studies of cognitive functioning. In our case 46% of patients were unable to participate due to poor performance status or other somatic factors.

The finding that a large proportion of patients are unable at all to participate in cognitive functioning studies additionally indicates an over-representation of patients with better cognitive functioning in research. From this point of view the cohort study design corresponds better to the everyday clinical practice with the patients with high grade glioma.

Patients’ self-assessments on a 1–10 scale did not correlate with the results of the tests used and probably should not be used for any assessment of cognitive functioning; we only found a weak correlation between self-assessment on a 10-point scale and objective assessment. With otherwise different methodology, foreign studies also came to similar results - there is no or weak correlation between subjective assessment and psychological tests.^29^

The limitation of our study is lack of data on cognitive functioning prior to surgical treatment. Thus, in the study we did not include eventual differences between patients with IDH1 mutated and wildtype tumours, which may be present even before surgery^19,30^, which would also be important in the light of research findings regarding the different dynamics of cognitive decline after surgery.^31^

Another point worth mentioning is that several papers showed that epilepsy and the use of antiepileptics is an important factor of neurocognitive functioning. But in our sample, the use of antiepileptics could not be analysed as virtually every patient has received them following surgery even those without history of seizures; though in these cases they were weaned from antiepileptics at the beginning of oncological treatment.

Our study took place during the coronavirus pandemics. Despite this, oncological treatment was not interrupted nor delayed, but in our study, it was connected with the increase of patients refusing to participate and with longer time from surgery to the start of treatment due to infections.

It is worth to mention that targeting this population is beyond single institution capabilities. While the cohort study corresponds better to the clinical practice, on the other hand the low number of the mutations, especially in IDH1, is hampering the statistical analysis. When conducting our study, we noted a distinctive lack of prospective data regarding patients in suboptimal performance status, thus overestimating cognitive functioning of high-grade glioma patients. Even as we observed the patients in WHO performance status of 2 the number of cognitive patients rose markedly.

It is true that the single centre study is limited in its power to demonstrate effect the genetic and molecular changes exert on cognitive functioning in real life scenarios, the reason being rightly, that outside the trials where cognitive functioning is one of secondary outcomes to survival and time to progression where treatment compliance effectively excludes patients with more pronounced impairments and in reality the cognitive impairment is more widespread in our patients than reported previously, which should be taken into account in designing further studies.

Our study has finished recruiting, but the longitudinal part of the follow up is continuing, thus giving us the chance to determine the impact of genetic and epigenetic changes on cognitive functioning in patients surviving longer and maybe even determining if cognition can be used as predictive marker for progression.
